# Circulating matrix metalloproteinase MMP-9 and MMP-2/TIMP-2 complex are associated with spontaneous early pregnancy failure

**DOI:** 10.1186/1477-7827-11-2

**Published:** 2013-01-15

**Authors:** Ritva Nissi, Anne Talvensaari-Mattila, Vesa Kotila, Maarit Niinimäki, Ilkka Järvelä, Taina Turpeenniemi-Hujanen

**Affiliations:** 1 Department of Obstetrics and Gynecology, Oulu University Hospital, Kajaanintie 52A, Oulu, 90220, Finland; 2 Department of Electrical Engineering, Division of Mathematics, University of Oulu, Finland; 3 Department of Oncology and Radiotherapy, Oulu University Hospital, Kajaanintie 52A, Oulu, 90220, Finland

**Keywords:** MMP-2, MMP-9, TIMP-1, TIMP-2, Pregnancy, Pregnancy loss, Serum marker, Placentation

## Abstract

**Background:**

Trophoblast cell (CTB) invasion into the maternal endometrium plays a crucial role during human embryo implantation and placentation. This invasion is facilitated by the activity of matrix metalloproteinases, which are regulated by tissue inhibitors of MMPs (TIMPs).

**Methods:**

This study compares the serum levels of MMP-9, MMP-2/TIMP-2 complex, TIMP-1 and TIMP-2 in 129 patients with ongoing pregnancy (n = 40) or spontaneous early pregnancy failure (n = 89).

**Results:**

MMP-9 was markedly (p < 0.0001) elevated in missed abortions, as was MMP-2/TIMP-2 complex (p < 0.0005). However, the serum levels of TIMP-1 and TIMP-2 were markedly elevated (p < 0.0001) in ongoing pregnancies.

**Conclusions:**

Human placentation is mediated by fetal trophoblastic cells that invade the maternal uterine endometrium. Trophoblast invasion requires a precisely regulated secretion of specific proteolytic enzymes able to degrade the endometrial basement membrane and extracellular matrix. The elevated levels of MMP-9 and MMP-2/TIMP-2 complex may play a role in spontaneous termination of pregnancy.

## Background

Matrix metalloproteinases (MMPs) can be produced by numerous cell types. In normal adult tissue they are almost undetectable by immunohistochemistry, but injuries and pregnancy, for example, elevate their protein deposition. MMPs have the ability to break down several proteins of the extracellular matrix (ECM) and they participate actively in remodeling the ECM by degrading important matrix scaffold macromolecules. Together with their tissue inhibitors (TIMPs) they form a balance to maintain normal early pregnancy and placental development.

The gelatinases MMP-2 and MMP-9 are especially involved in successful cytotrophoblast invasion in early pregnancy as they are considered key enzymes in degradation of basement membrane, which mainly consists of type IV collagen [1,2]. Tissue inhibitors for MMPs, such as TIMP-1 and TIMP-2, regulate protease activity.

MMP-2, MMP-9, TIMP-1 and TIMP-2 have been localized in the placental bed using immunohistochemistry and *in situ* hybridization. Immunoreactivity for MMP-2 was detected in both decidual cells and extravillous trophoblasts (EVT), but MMP-9 staining was only observed in areas with abundant EVT [1-4]. In early gestation weeks (weeks 6 and 7) the secretion of MMP-9 in placental bed is very low, but the secretion increases gradually after week 8, and in week 11 the cells produce a large amount of MMP-9 [1]. In contrast, biosynthesis of MMP-2 is significantly higher in the early stages of the pregnancy [3]. MMP-2 has been suggested to be the key regulator of trophoblast invasion in early pregnancy [4]. MMP-2 is localized in the placental bed during early pregnancy and it is dominant over MMP-9 on the trophoblasts of 6–8 weeks of gestation [5]. During labor, MMP-9 is mainly responsible for gelatinolytic activity in the membranes. Trophoblasts of the human placenta can differentiate into extravillous trophoblasts (EVT) with invasive properties. Proteolytic enzymes such as MMP-2 and MMP-9 are essential for the invasion of EVT cells into endometrial stroma [5].

In most previous studies the MMP levels have been studied *in vivo* by using animal models or tissue samples, but the human serum changes of MMPs and TIMPs in pregnancy have only been defined in few studies. An earlier study showed alterations in the concentrations of proMMP-9 and TIMP-1 in plasma or serum and urine of pregnant women experiencing term or preterm uterine contractions [6]. The aim of the present study was to compare the serum levels of MMP-9, MMP-2/TIMP-2 complex, TIMP-1 and TIMP-2 in 129 patients with ongoing pregnancy (n = 40) or spontaneous early pregnancy failure (n = 89) in order to evaluate the potential roles of matrix-degrading proteases MMP-2 and MMP-9 in the process of early pregnancy failure.

## Methods

The study was conducted in Oulu University Hospital at the department of Obstetrics and Gynecology from 4 February 2003 to 8 April 2005. 129 patients were enrolled in this study, which was approved by the ethics committee of the Northern Ostrobothnia Hospital District. Before participation, informed consent was taken from all patients.

The patients were divided into three groups. Group 1 included women with anembryonic pregnancy (n = 42). Group 2 comprised patients with incomplete spontaneous abortion or missed abortion with visible fetus (n = 47). Group 3 consisted of women with uneventful ongoing pregnancy (n = 40). The gestational age was measured by ultrasound. The patients with anembryonic pregnancy or aborted pregnancy sought treatment for abnormal bleeding and were examined on the same day when the bleeding started. The patients were healthy and 7–11 weeks pregnant. Outcome measures assessed differences in MMP-9, TIMP-1, TIMP-2 and MMP-2/TIMP-2 complex serum levels.

Venous blood samples were collected after ultrasound examination. Sera were obtained by centrifugation without using any artificial coagulation activator and stored frozen at −20°C until analysis for this study.

The concentrations of MMP-9, TIMP-1, TIMP-2 and MMP-2/TIMP-2 complex in the serum of the study patients were determined by enzyme-linked immunosorbent assay (ELISA). ELISA assays were performed on 8-well EIA/RIA microtiter plates (Corning Inc., Corning, NY, USA) using standard protocols [7]. Standard samples were included in every plate and the standard curves were required to be similar in each lot. All measurements were performed in duplicate. The wells were coated overnight at 4°C with a specific monoclonal antibody provided by SBA Sciences, Oulu, Finland (code DB-102 for TIMP-1, code T2-101 for TIMP-2 and MMP-2/TIMP-2, code Ge-213 for MMP-9). Following coating, diluted serum samples and standards for TIMP-1, TIMP-2 and MMP-2/TIMP-2 complex were incubated for 60 minutes, or overnight in the case of MMP-9. Non-specific binding was blocked with phosphate-buffered saline containing 1% bovine serum album (BSA-PBS). The wells were washed thoroughly before each stage of the procedure, in the first phase with PBS and in the later stages with PBST (0.05% Tween 20 in PBS). The bound proteins were detected with polyclonal antibodies against each of the analyses (anti-TIMP-1, code DB-205 for TIMP-2, code DB-202 for MMP-2/TIMP-2 complex, code DB-209 for MMP-9) (SBA Sciences, Oulu, Finland). A peroxidase conjugated anti-chicken antibody (Chemicon International, CA, USA) was used to detect the bound polyclonal antibody, and an OPD solution (*o*-phenylenediamine dihydrochloride, P-1526; Sigma, Steinheim, Germany) was used to visualize the peroxidase conjugate. The reaction was stopped with 1.8 M H2SO4. Color formation was measured at 492 nm with a microplate reader (Anthos Reader 2001; Anthos Labtec Instruments, Walls, Austria) using the Windows-based control and evaluation software for Rosys Anthos microplate readers (Anthos Labtec Instruments). The sensitivity of the assays was 2 ng/mL for MMP-9, 1 ng/mL for TIMP-1, 2 ng/mL for TIMP-2 and 2 ng/mL for MMP-2/TIMP-2 complex.

The laboratory data was analyzed statistically using Matlab 7.0.4.365 for Windows. A Shapiro-Wilk normality test was used to determine the normality of the samples in different patient groups. The values were not normally distributed. Hence, a nonparametric Mann–Whitney U test was used to compare the median values between groups against the null hypothesis that the samples come from the same distribution. The significance level was considered p < 0.005.

The normal serum levels for these enzymes have not been established. Therefore we analyzed the serum levels of MMP-9, TIMP-1, TIMP-2 and MMP-2/TIMP-2 complex on 27 volunteers (healthy non-pregnant women).

## Results and discussion

All patients were healthy and 7–11 weeks pregnant. The mean age of the patients was 30 years in missed abortions and anembryonic pregnancies, and 27 years in ongoing pregnancies (p = 0.02). The duration of gestation was shorter in women having ongoing pregnancies (mean 55.1 days) than in women with anembryonic pregnancy (mean 72.4 days) or missed abortion/spontaneous miscarriage (mean 77.9 days, p < 0.001).

There were no other differences in previous abortions or smoking habits between these patient groups. There were no clinical signs or symptoms for infection in any of the patients.

**Figure 1:**
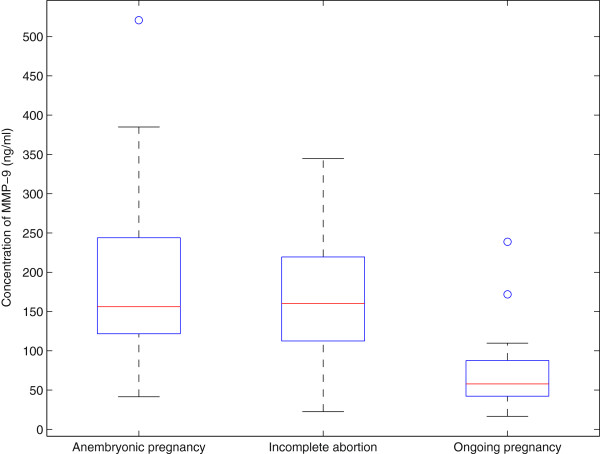
**The box-and-whisker diagrams of MMP-9 serum levels in the three patient groups. **The box represents the values from the lower quartile Q1 to the upper quartile Q3 while the red line marks the position of the median Q2. If a value exceeds Q3 or falls below Q1 by more than 1.5 × (Q3-Q1), it may be considered an outlier and is marked by a circle. The whiskers indicate the extent of the rest of the data. Three fourths of the measured values in the groups anembryonic pregnancy and incomplete abortion exceed the values in the group ongoing pregnancy, except for the outliers.

The median serum level of MMP-9 was 156 ng/mL in anembryonic pregnancies, which was comparable to that in the group of incomplete abortions (160 ng/mL). In ongoing pregnancies the level was 58 ng/mL, which is comparable to the values in healthy volunteers (63 ng/mL). Figure 1 demonstrates the shift of medians in anembryonic pregnancies and incomplete abortions compared to ongoing pregnancies. Statistical analysis showed that the differences between these groups are highly significant (p = 2 × 10^-10^ and p = 5 × 10^-10^, respectively).

**Figure 2:**
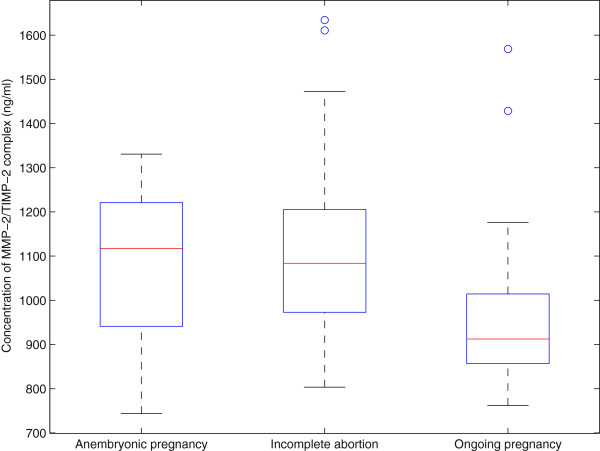
**The box-and-whisker diagrams of MMP-2/TIMP-2 complex serum levels in the three patient groups. **Aside from the outliers, there is a noticeable shift upwards in the groups anembryonic pregnancy and incomplete abortion compared to ongoing pregnancy.

**Figure 3:**
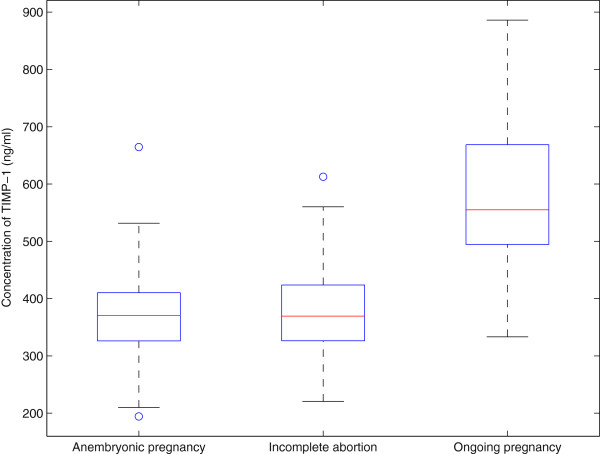
**The box-and-whisker diagrams of TIMP-1 serum levels in the three patient groups. **Nearly all measured values in the groups anembryonic pregnancy and incomplete abortion fall below the median in the group ongoing pregnancy.

**Figure 4:**
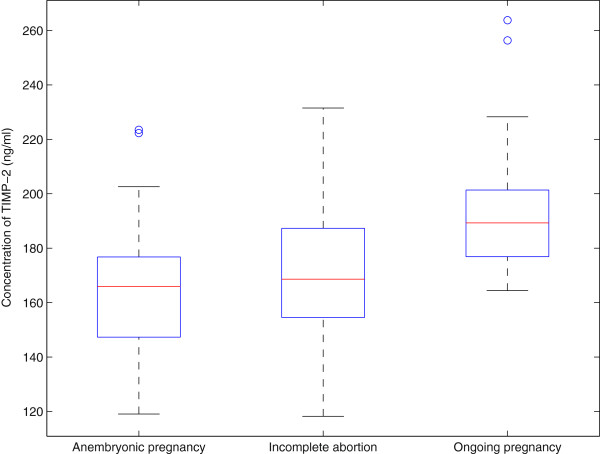
**The box-and-whisker diagrams of TIMP-2 serum levels in the three patient groups. **Again, a noticeable shift downwards can be observed in the groups anembryonic pregnancy and incomplete abortion.

The MMP-2/TIMP complex determines invasiveness [4], and the concentrations of the circulating MMP-2/TIMP-2 complex were investigated. Increased expression (p = 4 × 10^-4^ and p = 2 × 10^-5^) was seen in groups of anembryonic pregnancies (median 1117 ng/mL) and incomplete abortions (median 1084 ng/ml) when compared to ongoing pregnancies (median 912 ng/mL). The average serum level on healthy volunteers was 625 ng/mL. The results are shown in Figure 2. When comparing the levels of immunoreactive serum TIMP-1 protein, statistically significant differences were also seen between the study groups (p < 10^-10^), but in the opposite direction. The highest concentrations were seen in ongoing pregnancies (median 555 ng/mL), whereas in anembryonic pregnancies the TIMP-1 level was 371 ng/mL, which was also comparable to the concentrations seen in incomplete abortions (median 370 ng/mL), as shown in Figure 3. Similarly, the TIMP-2 levels were highest in ongoing pregnancies (median 189 ng/mL) when compared to anembryonic pregnancies (p = 8 × 10^-7^, median 166 ng/mL) and incomplete abortions (p = 3 × 10^-5^, median 169 ng/mL), as shown in Figure 4. In healthy volunteers the average values were 462 ng/mL for TIMP-1 and 324 ng/mL for TIMP-2.

On the basis of the present data we speculate that the increase in serum levels of MMP-2/TIMP-2 complex and MMP-9 as well as their inhibitors TIMP-1 and TIMP-2 could reflect the altered architecture of the extracellular matrix during pregnancy.

Successful pregnancy is dependent upon invasion of trophoblasts into the decidua at implantation. Blastocyst implantation that begins with the attachment of the trophoblast to the uterus and terminates with the formation of placenta is a complex series of events. Many studies have reported that MMP-9 is the key enzyme in the trophoblast invasion, but there are studies suggesting that MMP-2 appears earlier than MMP-9. Thus the invasive capacity of trophoblasts may be regulated by MMP-2 [4-6,8]. Interestingly, TIMPs have also been shown to be able to stimulate proliferation and play a direct role in the development of the intrauterine structures [9,10]. The expression of MMP-2 and MMP-9 varies significantly throughout the first trimester of pregnancy [1]. The difference in the duration of gestation may have caused some bias in the present study and needs to be acknowledged by further research.

MMPs have many roles in physiological processes, such as proliferation, survival, migration and morphogenesis. Co-expression of MMPs and TIMPs in trophoblasts suggests that the invasive and lytic properties of a cell could be dependent on the balance between MMPs and TIMPs [8]. Both MMPs and TIMPs are involved in tissue remodeling that accompanies the rapid growth and structural changes of the tissues. In the first trimester the invasive behavior of throphoblast cells is possibly due to the ability of EVT to secrete MMPs since TIMP should inhibit their invasiveness. TIMP-1 and TIMP-2 are considered to be responsible for protecting vessels and maintaining blood vessels’ integrity as inhibitors of MMPs [8]. The role of TIMP-1 might be more distinct, since it has also been shown to inhibit other metalloproteinases besides MMPs [4-6,8].

Normal human development is a complex event involving a series of interactions between the developing embryo and the receptive endometrium ultimately leading to successful establishment of pregnancy. The blockade of the biological progesterone effect was associated with an increase in the activity of both collagenases in medical termination of pregnancy, for example [11].

An inflammatory-like response takes place in the cervix in women with both symptomatic and silent miscarriage. In recurrent spontaneous abortion 50% of cases remain unexplained and unresolved. Recent data show that the immune system in a late-stage miscarriage is completely different from that in an early-stage miscarriage. Proper function of the immune system and cytokines is mandatory for a successful pregnancy outcome [12].

## Conclusions

Our findings suggest that MMP-9 and MMP-2/TIMP-2 complex are involved in intra-uterine tissue remodeling during the establishment of pregnancy. MMPs have been shown to affect cell survival and proliferation both positively and negatively by regulating survival signals generated by specific adhesive events [9,10,13,14]. However, the study groups differed significantly in terms of the age of the women and the duration of gestation, and thus the results should be interpreted with caution.

To the best of our knowledge, this is the first study to examine the serum levels and activity of MMPs and TIMPs in women with ongoing or spontaneously terminated pregnancy in the first trimester. As pregnancy is a dynamic environment and MMPs contribute to their constant remodeling throughout the pregnancy, further research in this field is warranted. The detection of the normal and pathological patterns of serum MMPs and TIMPs could possibly be used as a prognostic test for the risk of miscarriage or preterm birth in the future.

## Competing interest

The authors declare that they have no competing interests.

## Authors’ contributions

MN, IJ and ATM collected the material. VK performed the statistical analysis. ATM, TTH, RN and MN drafted the manuscript. TTH was resposible of the laboratory work. All authors read and approved the final manuscript.
